# Social encapsulation of parasite eggs by honeybee colonies

**DOI:** 10.1038/s41598-026-40183-5

**Published:** 2026-03-07

**Authors:** Francesca M. Grech, Anna Papach, Aura K. Palonen, Andrew Brown, Anthony Abbate, Geoffrey R. Williams, Peter Neumann

**Affiliations:** 1 Institute of Bee Health, Vetsuisse Faculty, University of Bern, Bern, Switzerland; 2 Bee Center, Department of Entomology & Plant Pathology, Auburn University, Auburn, AL USA

**Keywords:** Parasite, Small hive beetle, Honeybee, Behavioural defence, Encapsulation, Ecology, Ecology, Zoology

## Abstract

Eusocial insect colonies can choose different behaviours to combat the same parasites. While honeybee (HB), *Apis mellifera*, colonies remove eggs and larvae of parasitic small hive beetles (SHB), *Aethina tumida*, they encapsulate adults in propolis. It is currently unknown whether there is flexibility in this system. Here, we show encapsulation of eggs by colonies as an alternative to removal. Oviposition sites with or without SHB eggs were introduced into HB field colonies of mixed European origin. After 24 h, sites were removed to measure the propolised area and to quantify the remaining eggs. Further, SHB egg laying depth, and HB worker proboscis length and thickness, were measured. Even though the proboscises were long enough to reach most eggs, the number of eggs before and after introduction did not differ. Instead, HB used more propolis on sites with eggs compared to controls. This suggests decision making by colonies for either egg removal or encapsulation and demonstrates considerable flexibility in social immunity. Such flexibility probably contributes to eusocial insect resilience and calls for efforts to understand colony decision making.

**Supplementary Information:**

The online version contains supplementary material available at 10.1038/s41598-026-40183-5.

## Introduction

Eusocial insect colonies can be regarded as superorganisms, consisting of individual animals analogous to cells in the metazoan^1,2^. Due to enhanced parasite transmission^3^, these superorganisms have evolved various social immunity traits^4,5^. In honeybee colonies, *Apis mellifera*, well known examples of such social immunity are grooming of nestmates and removal of infected brood^6,7^(hygienic behaviour). Furthermore, colonies can also encapsulate invaders with propolis, which is tree resin collected by the bees^8^.

Flexibility in social immunity appears to be crucial for colony survival, enabling them to adapt to changing environmental conditions and pathogen threats^9^, especially in the case of novel threats such as invasive species^10^(e.g., mite *Varroa destructor*). However, in most cases, such flexibility is poorly understood. This holds true for small hive beetles (SHB), *Aethina tumida*, which are parasites of honeybee, *A. mellifera*, colonies native to sub-Saharan Africa^11–13^. In their native range, SHB are generally considered a minor pest^11,12,14^. However, since 1996, the SHB has spread across various continents and countries, where it can cause serious damage to apiculture^13,15^. SHB lay eggs in typical clusters in cracks and crevices of the nest, directly in pollen or brood cells, or around the nest periphery, which usually hatch within three days^11,14^. Most damage to host colonies is done by the emerging SHB larvae which feed on honey, pollen, and bee brood, sometimes leading to the full structural collapse of the entire nest^11,15^. Honeybees can use various social immunity traits against SHB, e.g., encapsulating adults in propolis^16–18^and removal of eggs and larvae^11,19,20^. For example, 34% of SHB eggs that were placed inside African honeybee colonies were removed within 24 hours^19^. Since both African and European-derived honeybees show the same behaviours towards SHB, quantitative differences in a range of host behaviours may underlie their differential impact in endemic and invasive ranges^15^. Given that honeybee workers use their proboscis to remove SHB eggs laid in cracks^19^, the maximum SHB egg laying depth vs. proboscis length may determine the efficacy of this social immunity trait. However, neither the removal rates of SHB eggs nor the possible relation between honeybee worker proboscis length/SHB egg laying depth has been estimated for European-derived honeybees. Furthermore, it is not known whether there is flexibility in the social immune response of honeybee colonies towards SHB, i.e., encapsulation of eggs has not been reported yet.

Here, we estimated for the first time the removal of SHB eggs by European-derived honeybee colonies. In addition, SHB ovipositor length, egg-laying depth, and the length and thickness of the honeybee worker proboscis were measured to determine whether the SHB can push eggs out of host reach. Since honeybees use propolis against adult SHB^16^, and stingless bees use resin against all SHB life stages^21^, the possibility for encapsulation of eggs was also examined. In addition, colony strength parameters were recorded to test for possible impact on egg removal or propolis usage.

## Results

### Egg removal and encapsulation behaviour

The egg removal by European-derived honeybees was evaluated by placing sites (two microscope slides glued together with “Elmer’s Clear School Glue”, spaced out with inserted cover slips on both ends) with SHB eggs into 10 field colonies for 24 h. A total of 12 test sites were excluded from the analyses because accurate egg counting post-retrieval was not feasible. In total, the egg removal rate was analysed using 38 oviposition test sites with eggs, and 7 control sites without eggs. After 24 h of placement in the 10 colonies, a median percentage of 100% (SE = 0.35, 91.3–100%) of SHB eggs remained. There were differences in encapsulation behaviour with the treatment sites containing SHB eggs [*N* = 50] showing a significantly higher propolised length along the sites (median = 52.6 mm, SE = 6.9, 0–195 mm) compared to the control sites [*N* = 50] (median = 25.0 mm, SE = 5.29, 0–142 mm; *W* = 1674.5, *P* < 0.002).

### Morphometrics: small hive beetle egg-laying depth and ovipositor length, honeybee worker proboscis length and thickness

**Fig. 1:**
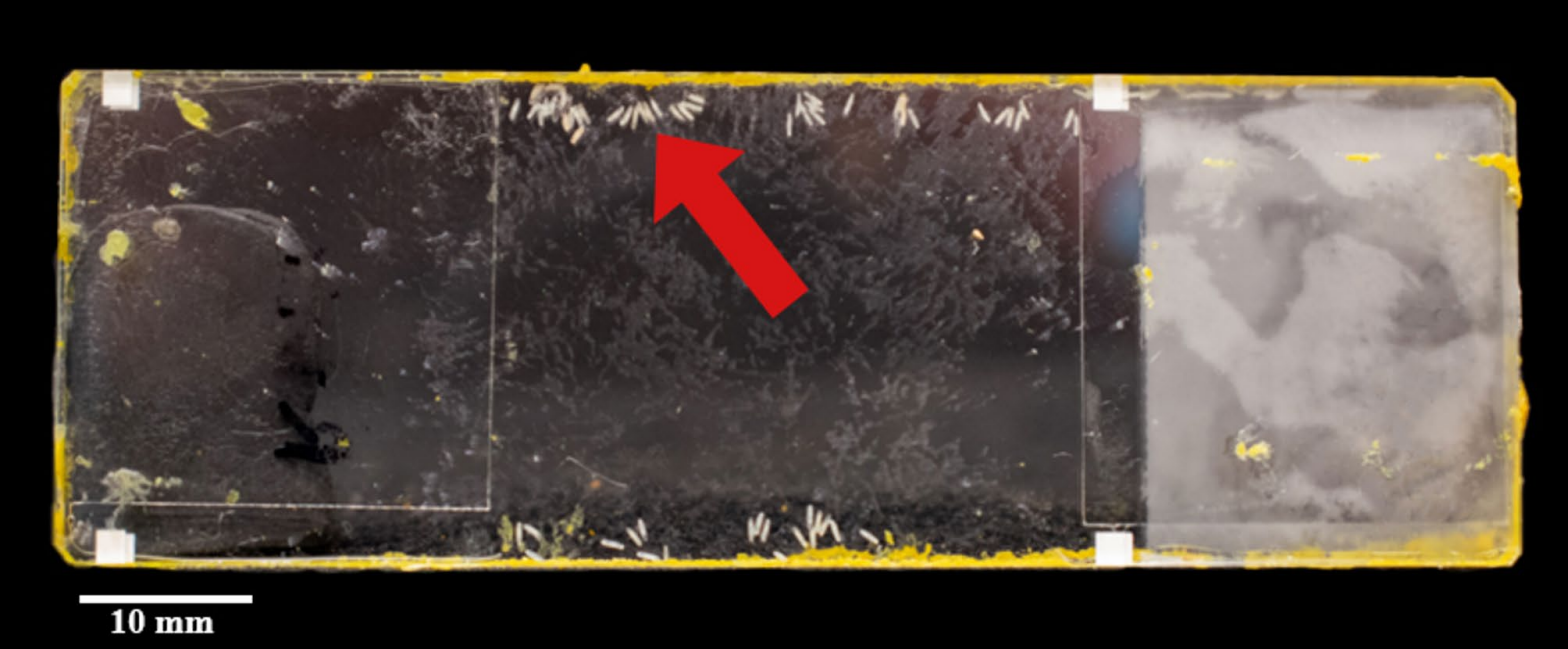
Oviposition site with small hive beetle, *Aethina tumida*, eggs after retrieval from a honeybee, *Apis mellifera*, colony. The site consists of two microscope slides separated with a cover slip on each end and glued together. The eggs were laid in the gap between the two slides (red arrow indicating egg cluster). The gap between the slides was propolised by honeybees (yellow area on the margins of the microscope slides).

**Fig. 2:**
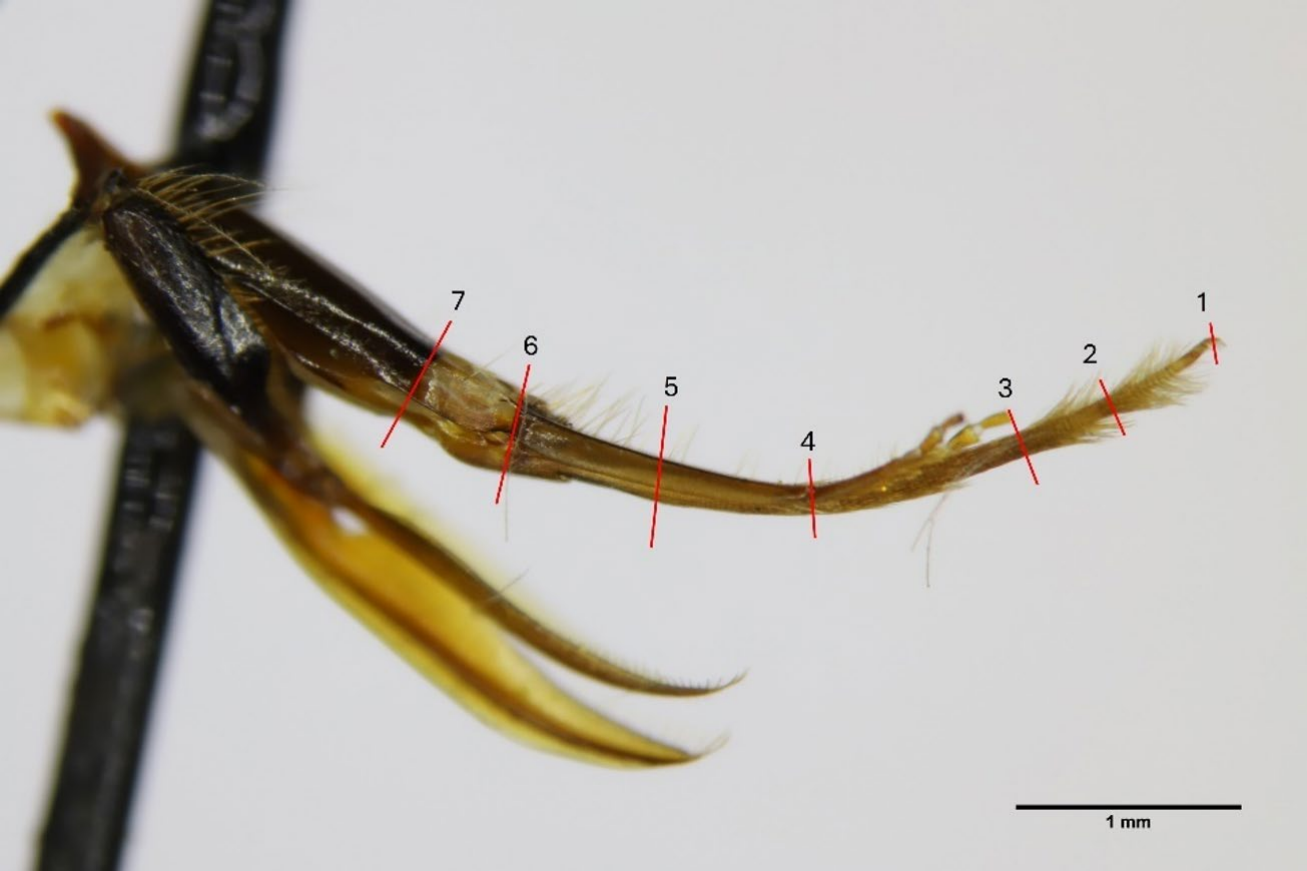
Lateral view of the proboscis of an adult honeybee, *Apis mellifera*, worker. Seven points were the thickness measurements were taken are shown: 1 - middle of labellum, 2 - midpoint between labellum and apex of labial palpus on glossa, 3- apex of labial palpus on glossa, 4 - apex of first segment of labial palps, 5 - midpoint between apex and base of first segment of labial palps, 6 - base of first segment of labial palps and 7 - where the black/brown line ends on prementum.

**Fig. 3:**
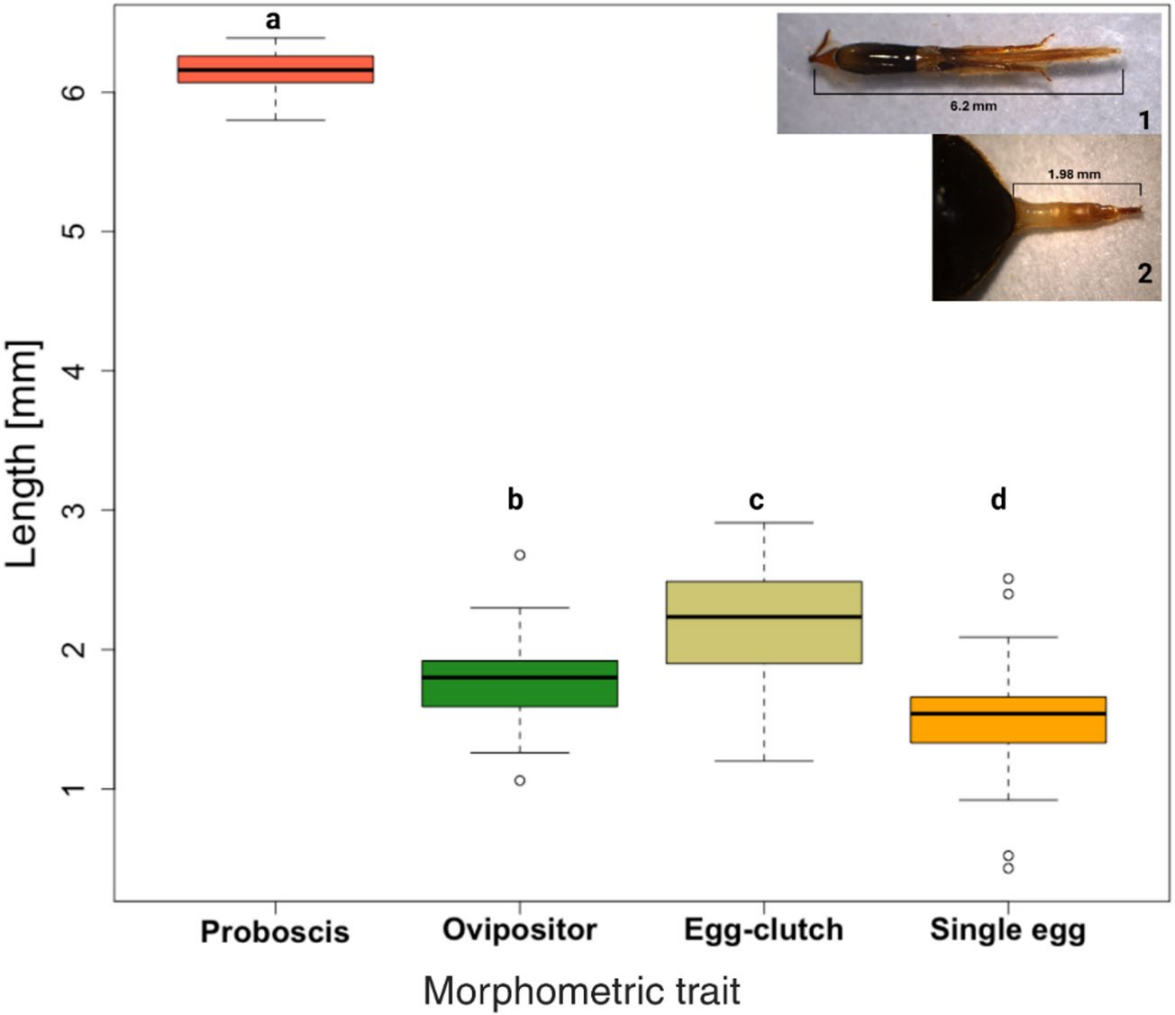
Proboscis length of the honeybee workers, *Apis mellifera*, (postmentum to the apex of the glossa (top right corner 1), ovipositor length of female small hive beetles, *Aethina tumida* (caudal tip of the abdomen to the apex of the ovipositor including the gonostylus) (top right corner 2) and egg-laying depth of egg-clutches and single eggs of *A. tumida*. The letters above the boxes indicate significant differences between the groups (one way ANOVA followed by a TukeyHSD, *P* < 0.001).

There were significant differences between the proboscis length, the length of the ovipositor, egg-laying depth of egg-clutches and single-laid eggs (F(3,276) = 5156, *P < 2e-16*). The proboscis length of honeybees (mean = 6.16 mm, SD = 0.13, Figs. 1,2) was significantly longer than the ovipositor length of SHB (mean = 1.77 mm, SD = 0.25; *P* < 0.001), the maximum egg-laying depth of the egg clutches (mean = 2.19 mm, SD = 0.41; *P* < 0.001; Fig. 3) and the single laid eggs (mean = 1.51 mm, SD = 0.42; *P* < 0.001, Fig. 3). Moreover, the egg-laying depth of the egg clutches was higher than the length of the SHB ovipositor (*P* < 0.001) and the depth of the single laid eggs (*P* < 0.001), while the length of the SHB ovipositor was greater than the depth of the single laid eggs (*P* < 0.001). However, the thickness of the honeybee worker proboscis varied along its length (Supplementary Table 1) and only part of it until the apex of labial palpus on glossa was able to fit into the oviposition site gap (no difference between the size of the oviposition site gap and the thickness at the apex of the labial palpus on glossa; *P* = 0.091). The size of the oviposition site gap was around 0.16 mm.

### Propolised length and colony phenotypes

A total of *N* = 100 oviposition sites [*N* = 50/treatment] was used to construct a least-squared model. The presence of small hive beetle eggs (*T* = 3.026, *P* = 0.003) was found to be a significant predictor of propolised length [mm], resulting in $\:\approx\:$ 1.9 mm more propolis.

**Fig. 4:**
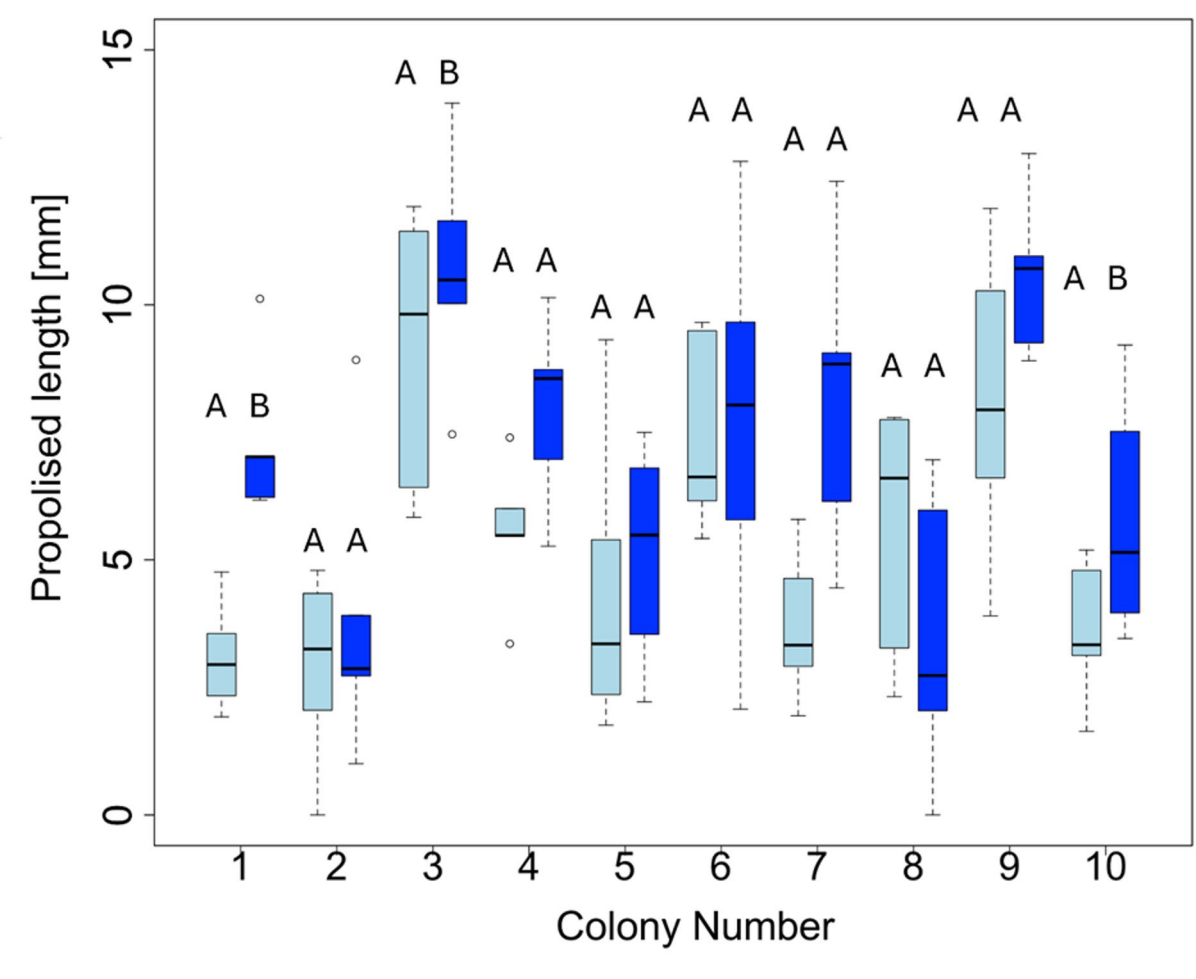
Boxplots of propolised length by honeybees, *Apis mellifera* from different hives (colonies) [*N* = 10] on oviposition sites without (control; light blue colour) and with small hive beetle, *Aethina tumida* eggs (treatment, dark blue colour). Five number summaries are displayed in each boxplot: minimum, first quartile, median, third quartile, and maximum values. Data displayed are square root transformed, and pairwise t testing, with Bonferroni adjustment, was done. Letters on top of each boxplot indicate statistical difference (*P* < 0.05) between control and treatment for each hive.

A significant difference in propolised length was calculated (F (10,89) = 8.536, *P* < 0.001, multiple R^2^ = 0.4896) depending on the hive (colony) origin (i.e., genetic differences). The presence of eggs (= Treatment) was a significant predictor for the additional amount of propolis used in a hive (β = 1.72, *P* < 0.001). For each of the individual hives, both treatment [*N* = 50] and control sites [*N* = 50] were included, resulting in various hives statistically significantly predicting propolised length, namely Hive 1 (= Intercept; β = 4.35, *P* < 0.001), Hive 3 (β = 4,69, *P* < 0.001), Hive 6 (β = 2.36, *P* = 0.03) and Hive 9 (β = 4.12, *P* < 0.001); Fig. 4; Supplementary Table 2).

None of the measured colony phenotype parameters – population (number of bees, β = −0.004094, *P* = 0.447), brood (β = 0.536656, *P* = 0.5148), honey (β = 0.550629, *P* = 0.0767), pollen (β = −0.052996, *P* = 0.8912) – could explain the propolised length [mm] (F (4,6) = 5.47, *P* = 0.03344, Multiple R^2^ = 0.7848). However, the stepwise algorithm, which was conducted on all Liebefeld colony parameters, resulted in honey as a significant predictor for the propolised length [mm] in the colonies (β = 0.37147, *P* < 0.001; F (1,9) = 28.14, *P* < 0.0005, multiple R^2^ = 0.7577). A coefficient of 0.37147 reflects an increase of ca. 4 mm of propolis per additional 1dm^2^of honey present in a colony.

## Discussion

The data show for the first time encapsulation of parasite eggs by honeybee colonies as an alternative to egg removal, supporting the notion of flexible social immunity in eusocial insect colonies. The length of the measured honeybee proboscises was significantly larger than the ovipositor of SHB, and significantly longer than the maximum egg-laying depth of egg-clutches. However, the thickness of the proboscis was a limiting factor preventing the bees from reaching all eggs. Nevertheless, almost all SHB eggs remained after 24 h in the tested colonies of European-derived honeybees, indicating the absence of or at least no detectable egg removal. Instead, honeybees used propolis to seal the gaps on the oviposition sites and propolised significantly larger areas on slides with eggs compared to the empty control sites. There were differences between the colonies in the propolised length suggesting genetic variance for this trait. The propolised length also positively correlated with the amount of honey present in the colonies. The present data contrast with Africa, where the protected SHB eggs were not encapsulated, but instead quickly removed by the honeybees (> 30% in 24 h)^19^. These striking differences demonstrate considerable flexibility in social immunity, calling for a better understanding of respective colony decision-making.

Insects are known to use different strategies to protect their eggs, among which, laying eggs in egg clusters is one of the common mechanisms^22^. It can provide benefits, such as protection against desiccation^23^or by protecting and increasing the chances of survival of the eggs in the middle from predation and parasitism^24^. Our data show that when SHB females lay eggs in egg clusters, it allows them to increase the egg laying depth compared to those laid singly. Since they lay eggs in cracks and crevices, they increase the egg-laying depth by pushing and stacking eggs on top of each other, thus providing a better hiding place for their offspring. We also show that the honeybee proboscis is long enough to reach all eggs in the clusters, but the thickness of the proboscis was a limiting factor that prevented them from removing all of the eggs. Even though part of the proboscis was thin enough to reach the shallowest eggs, we have not detected any significant egg removal behaviour. This is in sharp contrast to findings reported for African honeybees, where 34% of SHB eggs were removed within 24 hours^19^.

Instead of egg removal, honeybees in the present study used propolis to seal the gaps on the sites, thus limiting the spread of larvae after the egg hatching. Use of propolis as social encapsulation is long known for SHB adults^16,17^. This study is the first to report the use of propolis by honeybees to encapsulate parasite eggs. It seems that honeybees show flexibility in their behaviour against small hive beetle eggs, and the choice of one strategy over the other may be governed by some intrinsic and extrinsic factors. In the present study, honeybees were not able to reach all of the eggs, and even though their proboscis was thin enough to reach some of the shallowest eggs, they decided to adopt a different defence strategy. Animal behaviour, including their interactions with parasites can depend on spatial or temporal environmental heterogeneity^25^. It is known that seasonality can influence host immunity^26,27^and recently, the attention has also been drawn to the importance of spatial variation in the promotion of different immune phenotypes^28,29^. Those factors can also explain our findings as propolis collection is a very energy-demanding task^30^and depends on several factors such as season (temporal variation), but more importantly presence of trees within the foraging distance (spatial variation). Indeed, the experiment was conducted in August-September (local summer), when bees are usually collecting more propolis. Moreover, the colonies used in the present study were in proximity to large, forested areas, providing ample trees within foraging distance. In contrast, the colonies used in Neumann and Härtel (2004) were in the savannah with possible limited access to tree sap^19^(i.e., very few trees within 5 km).

When talking about the importance of spatial and temporal environmental variation, one should also take into consideration the parasite load. Even though it is still poorly understood what drives SHB infestation levels, infestation levels of honey bee host colonies can be equally high in the endemic and invasive ranges^31^. Nevertheless, it may well be that the local infestation levels govern the chosen behavioural strategies against different life stages of SHB. As with any behaviour, there can be certain thresholds that will dictate the choice of the defence strategy in the given conditions. For example, a heavily infested colony of African honeybees will readily abscond^32^instead of sealing anything as it might be a more efficient way to survive this parasite under given infestation levels. Absconding induced by SHB was also reported in colonies of European-derived honeybee colonies^33,34^. When the infestation levels are low, egg removal or the use of propolis to encapsulate the eggs appear to be better options, and the honeybees can prefer one over the other judging by the other external (environmental) factors. While SHB infestation levels were not estimated in this study, adult beetles were observed in rather low numbers (< 50, not objectively quantified) in the colonies during the estimation of colony strength parameters. Further, no visible signs of comb damage were observed (reviewed by Neumann and Elzen 2004), suggesting that the displayed encapsulation behaviour is not a response to high SHB infestation levels and affirming the use of propolis as a defensive tactic.

In our study, we also estimated multiple colony strength parameters and correlated them with the propolised length along the introduced sites with and without SHB eggs. Interestingly, the amount of honey present in the colonies was the only factor positively correlated with the propolised length. It has been shown that certain honeybee strains that produce more propolis also tend to produce more honey^35^. This combined with the observation that certain colonies in our experiment used more propolis compared to others, suggests that there is a genetic component to this behaviour as there is one for resin foraging^30^. These findings also suggest that there are other factors than colony phenotype that are influencing the choice of defence strategy against parasites by honeybees.

## Conclusions

This work is the first report of the use of propolis by honeybees against SHB eggs. It seems as if honeybee colonies can choose between several different behaviours to combat this parasite^20^(egg removal, encapsulation, jettisoning, absconding, etc.) based on currently unknown cues. Therefore, it appears important to better understand respective colony decision making, i.e., which social immunity approach is chosen under given environmental as well as internal conditions. Overall, our findings highlight the flexibility of social insect colonies and the importance of the spatial-temporal dynamics of host-parasite relationships.

## Materials and methods

### Laboratory rearing

From August to September 2022, all experiments were conducted at the Bee Center, University of Auburn, AL, USA. Adult SHB were manually collected from naturally infested local honeybee colonies of mixed European origin (predominantly *A. m. ligustica*) at three apiaries using standard methods^36^. The SHB were sexed^14^and used to initiate laboratory rearing^36^. Transparent plastic containers [*N* = 23] were prepared with adult SHB [*N* = 112]. Within each container, one oviposition site (two microscope slides glued together with “Elmer’s Clear School Glue”, spaced out with inserted cover slips on both ends; modified from Neumann et al., (2013)^36^was added. SHB were fed *ad libitum* with a diet of honey-pollen in a ratio of 1:2^36^. A paper towel [30 × 30 mm] was placed on the bottom of the containers to regulate moisture. Upon daily inspections, the inside of each cup was manually misted with water. The containers were stored in complete darkness at 25 °C and 80% relative humidity^36^(RH).

### Egg removal and encapsulation behaviour

Egg-clutches on the sites were used (i.e., multiple eggs (≥ 5) laid in multiple rows (Fig. 1). Every 24 h, the oviposition sites were checked and removed from the containers when a minimum of two egg-clutches on the oviposition sites were visible. Sites without clutches were left for a further 24 h or discarded. All sites with egg clutches were cleaned with a wet paintbrush and marked. Afterwards, each site was individually photographed using a Nikon D5300 camera equipped with an AF-P DX 18–55 mm lens and then used for the field experiments.

To evaluate SHB egg removal, we followed protocols of Neumann & Härtel (2004)^19^. At one apiary, ten colonies were randomly selected for the collection of adult SHB. Sites were placed centred on top of the three innermost frames of the top box of the hives. One to three sites were placed simultaneously per hive. As a negative control for egg hatching, we selected a single empty hive within the apiary that contained a single frame in which the sites were placed [*N* = 7]. After 24 h, all the sites were retrieved from the hives (Fig. 1). Pictures of the sites were taken after retrieval in the same procedure and with the same equipment as described previously. Photographs of the sites taken before and after placement in the hives [*N* = 57] were analysed using Windows Photo Viewer to count the number of eggs. Hatched larvae were observed on some sites before placement [*N* = 8]; these larvae were counted and deducted from the number of larvae observed on those sites after the 24 h timeframe.

To evaluate the encapsulation behaviour, five identical control oviposition sites without eggs were placed per hive [*N* = 50], in addition to the sites containing eggs. The control sites were placed in the same colonies and removed after 24 h. At a later stage, the length of the propolised gap of the control sites and the sites with eggs used for the egg removal was measured [*N* = 100]. For this, the length on the oviposition site where the gap was sealed over the entire width with propolis was marked with a waterproof marker and scanned using a printer “Brother MFC-J5330DW” along with a measuring tape for size reference. Using the software “ImageJ 1.53t”^37^, the entire length of the marked lines on each site in the images was measured. With the scale function of the program, the same scale for all the scanned sites was applied.

### Morphometrics: small hive beetle egg-laying depth and ovipositor length, honeybee worker proboscis length and thickness

#### Egg-laying depth

Photographs of the oviposition sites obtained during the egg removal experiment [*N* = 20] were used. With the software “ImageJ 1.53t”^37^, measurements of the furthest depth of the eggs in a clutch was measured perpendicularly from the edge of the microscope slide to the closer pole of the furthest egg present in a clutch [mm]. Furthermore, the furthest depth of single eggs on the same site [mm] – not in a clutch – was measured using the same procedure as with egg-clutches. Additionally, we measured the size of the gap [mm] that was provided for egg laying.

#### Ovipositor length

Freshly collected female SHB from the same three apiaries [*N* = 72] were freeze-killed to facilitate measuring the ovipositor. The same procedure was repeated for one to three randomly selected females from each container of adult SHB [*N* = 44] prepared for the egg removal experiment, leading to a total of 116 female SHB. The measurements were taken under a “Leica S9D” stereomicroscope with a mounted camera “MC170 HD” and using the software “Leica LAS V4.12.0”^38^ after initial calibration. Measurements were taken in the median plane from the caudal tip of the abdomen to the apex of the ovipositor (including the gonostylus) [mm].

#### Proboscis length and thickness

Nurse bees were collected from five hives used in the previously mentioned experiments and used for the measurements. The collected bees were starved for 3 h and freeze killed. The proboscis of nurse bees [*N* = 91] was separated from the head and measured using a calliper under a “Leica S9D” stereomicroscope for better precision; measurements were taken from the beginning of the postmentum to the apex of the glossa [mm]. Additionally, 10 nurse bees were used to measure the thickness of the proboscis. The proboscis was removed and mounted on a #2 insect pin, with the basal portion secured using clear Elmer’s glue. A lateral image of each proboscis was captured to measure its thickness. These measurements were taken at seven locations shown in Fig. 2 using ImageJ. To ensure accuracy, the software was calibrated with a 1 mm stage micrometer slide containing 100 subdivisions. A known distance (1 mm) on the calibration slide was measured thrice to verify accuracy. The same magnification was used for both calibration and proboscis imaging to ensure consistency. Images were taken using a Canon EOS 6D Mark II camera mounted on a Leica M205c microscope.

#### Colony phenotype data

During the removal experiments, the colony phenotypes of the ten colonies were evaluated (comb areas covered with adult bees, honey, pollen, worker and drone brood areas) using the “Liebefeld method”^39^. This method consists of the frame-by-frame visual estimation of the above-mentioned colony phenotypes and allows quantifying them.

#### Statistical analyses

All data analyses and visualization were performed in R version 4.3.0^40^ with additional packages “ggplot2”^41^, “psych”^42^, “tidyverse”^43^, and “MASS”^44^. Both visual checks (i.e., quantile-quantile plots) and Shapiro-Wilk normality tests were performed when necessary to check data distribution. Normally distributed continuous data were represented as mean and SD or median, SE and range (minimum-maximum) if not normally distributed. Significance was set at p ≤ 0.05.

To evaluate the egg removal rate, the natural hatching rate of larvae was first determined using the median value of the number of eggs of the control group before and after placement, leading to a natural hatching rate of 11.47%. This value was then used to calculate the number of naturally hatched eggs on each site in the test group and after was deducted from the number of eggs on the treatment sites after the removal from the colonies. The egg removal rate of the treatment group was subsequently determined by calculating the difference in eggs before and after the 24-hour period with the corrected number of eggs.

To test for differences in the encapsulation behaviour, the propolised length of the treatment [*N* = 50] oviposition sites was compared with the propolised length of the control sites [*N* = 50], with a two-sample one-sided Wilcoxon rank sum test (alternative = greater).

The mean values and SD of the ovipositor and proboscis lengths, egg length and egg laying depth of single eggs and egg clutches were calculated. One-way ANOVA followed by Tukey HSD were used to test for the differences between the measurements: the proboscis length [*N* = 91], the ovipositor length [*N* = 116], the egg laying depth of egg-clutches [*N* = 38] and the depth of single-laid eggs [*N* = 35]. Differences in proboscis thickness at seven positions compared to the oviposition gap were tested using a one-way ANOVA followed by Dunnett’s post hoc test to compare each measurement to the control (oviposition site gap).

The influential value of various factors on the propolised length was further examined using the following parameters: presence of SHB eggs, Liebefeld colony assessment parameters (number of bees, brood [dm^2^], honey [dm^2^] and pollen [dm^2^]) as well as the propolised length per colony on treatment and control sites combined. Data were analysed with least-squared regression models, using either square-root transformed or “raw” untransformed data, using the function lm(), with a defined “Gaussian” error distribution. ANOVA results were reported, and pairwise t-testing with Bonferroni p-value adjustment was applied when appropriate. Lastly, for the model reporting influence of Liebefeld colony assessment parameters on the average propolised length [mm], reverse model selection was done using the stepAIC() function.

## Supplementary Information

Below is the link to the electronic supplementary material.

Supplementary Material 1

## Data Availability

The original data is publicly available at DRYAD data repository. [http://datadryad.org/share/LINK_NOT_FOR_PUBLICATION/Ncs_3J2au81vDQGSV6Cc7qhfdI1Wda4ma4Il9ChK2Ro](http:/datadryad.org/share/LINK_NOT_FOR_PUBLICATION/Ncs_3J2au81vDQGSV6Cc7qhfdI1Wda4ma4Il9ChK2Ro).
