# Supplementary material for: Social encapsulation of parasite eggs by honeybee colonies
Source: Sci Rep. 2026 Mar 7;16:12542. doi: 10.1038/s41598-026-40183-5 (PMC13087306; doi:10.1038/s41598-026-40183-5)
Supplement: Supplementary file 1 — Supplementary Material 1 [file 41598_2026_40183_MOESM1_ESM.docx]

Table 1. Mean values along with the standard deviations (SD) of measurements of the gap of the oviposition sites used for small hive beetle, Aethina tumida, egg laying and thickness of the honeybee, Apis mellifera, proboscis along its length are shown. Differences from the oviposition site gap (control) are shown as mean difference with 95% confidence interval (CI) and the corresponding P-value. Statistical comparisons were performed using a one-way ANOVA followed by Dunnett’s post hoc test.

| **Measurement** | **Mean ± SD [mm]** | **Difference vs. width of the oviposition site gap** | **95% CI** | ***P -value*** |
| --- | --- | --- | --- | --- |
| Width of the oviposition site gap | 0.16 ± 0.01 | - | - | - |
| Thickness at the middle of labellum | 0.037 ± 0.001 | -0.13 | -0.16 to -0.09 | <0.001 |
| Thickness at the mid point between labellum and apex of labial palpus on glossa | 0.1 ± 0.02 | -0.06 | -0.09 to -0.02 | 0.0013 |
| Thickness at the apex of labial palpus on glossa | 0.13 ± 0.03 | -0.03 | -0.07 to 0 | 0.0906 |
| Thickness at the apex of first segment of labial palps | 0.15 ± 0.02 | -0.01 | -0.05 to 0.03 | 0.9489 |
| Thickness at the midpoint between apex and base of first segment of labial palps | 0.2 ± 0.03 | 0.04 | 0 to 0.08 | 0.0332 |
| Thickness at the base of first segment of labial palps | 0.37 ± 0.03 | 0.21 | 0.17 to 0.24 | <0.001 |
| Thickness at the where the black/brown line ends on prementum | 0.37 ± 0.03 | 0.21 | 0.17 to 0.24 | <0.001 |

| **Variable** | **Coefficient** | **Std. Error** | **T-Value** | ***P*** **-value** |
| --- | --- | --- | --- | --- |
| (Intercept) | 4.35 | 0.82 | 5.31 | <0.001 |
| Hive 2 | -1.82 | 1.10 | -1.65 | 0.10 |
| Hive 3 | 4.69 | 1.10 | 4.26 | <0.001 |
| Hive 4 | 1.53 | 1.10 | 1.39 | 0.17 |
| Hive 5 | -0.35 | 1.13 | -0.31 | 0.76 |
| Hive 6 | 2.36 | 1.10 | 2.14 | 0.03 |
| Hive 7 | 0.74 | 1.10 | 0.68 | 0.50 |
| Hive 8 | -0.66 | 1.10 | -0.60 | 0.55 |
| Hive 9 | 4.12 | 1.06 | 3.90 | <0.001 |
| Hive 10 | -0.55 | 1.13 | -0.49 | 0.63 |
| Treatment (presence of eggs) | 1.72 | 0.49 | 3.48 | <0.001 |

**Table 2** Model summary results from a least-squared regression model (lm) of propolis content [mm] used by honeybees, *Apis mellifera* on sites with and without small hive beetle, *Aethina tumida,* eggs explained by hive origin (i.e.genetic differences) and presence/absence of eggs (control) or present with small hive beetle eggs [N=2]. All data were square root transformed. Model coefficients, standard errors, t-values, and p-values are displayed (ANOVA, F= 8.536, *P* < 0.001)
